# Stroma-Rich Hyaline Vascular Type of Castleman Disease: A Case Report and Literature Review

**DOI:** 10.7759/cureus.60435

**Published:** 2024-05-16

**Authors:** Ranim F Al Derbas, Sarah A Al Nafisi, Ahmad T Al Khiary, Fatimah S Al Ghamdi, Fadel Z Al Oatibi

**Affiliations:** 1 Pathology, Prince Sultan Military Medical City, Riyadh, SAU; 2 Pathology and Laboratory Medicine, Security Forces Hospital, Riyadh, SAU; 3 Pathology, King Faisal Specialist Hospital and Research Centre, Riyadh, SAU; 4 Nephrology, Prince Sultan Military Medical City, Riaydh, SAU; 5 Pathology, Security Forces Hospital, Riaydh, SAU

**Keywords:** paraneoplastic pemphigus, myasthenia gravis, stroma-rich, plasma cell, castleman disease

## Abstract

Castleman disease (CD) is a rare lymphoproliferative disorder characterized by abnormal lymph node enlargement. We present the first documented case of a stroma-rich variant of hyaline vascular Castleman disease in Saudi Arabia. A 24-year-old Saudi female known to have acetylcholine receptor antibody-positive myasthenia gravis (MG) presented with shortness of breath, oral thrush, and an acute myasthenia gravis exacerbation, necessitating intensive care unit (ICU) admission. During her hospitalization, she was found to have a large pelvic mass. The mass was surgically excised. The diagnosis of stroma-rich hyaline vascular Castleman disease was rendered after histopathological examination. The patient's symptoms improved after the surgery.

This case underscores the importance of considering Castleman disease in complex clinical presentations, especially in the context of autoimmune and paraneoplastic diseases. Recognition and timely intervention are crucial for patient management. Additionally, the report adds to the global literature on Castleman disease, emphasizing the need for further research into its clinical manifestations and associations.

## Introduction

Castleman disease (CD), first described by Benjamin Castleman in 1956, represents a spectrum of rare lymphoproliferative disorders characterized by hyperplastic lymphoid follicles within a single or multiple lymph nodes or extranodal sites [1-3]. Based on the clinical presentation, the CD can be classified into two major categories: unicentric (localized) and multicentric (systemic) variants [2,4]. The localized form of unicentric Castleman disease (UCD), typically asymptomatic, involves a solitary lymph node and is often incidentally discovered [5]. In contrast, the multicentric Castleman disease form (MCD) is associated with systemic symptoms, including fever, weight loss, night sweats, lymphadenopathy, and various laboratory abnormalities [5]. Histologically, CD exhibits three primary subtypes: hyaline vascular Castleman disease (HVCD), plasma cell Castleman disease (PCD), and mixed. The HVCD subtype is characterized by small lymphoid follicles, regressed germinal centers, and hyalinized blood vessels. The PCD subtype is marked by prominent interfollicular plasma cell infiltrates, and the mixed subtype combines features of both HVCD and PCD variants [6]. In 1993, Danon et al. described a variant of HVCD with florid proliferation of fibroblast-like cells in the interfollicular area. It was called a stroma-rich variant of Castleman disease of hyaline-vascular type (SR-HVCD) [7]. One of the intriguing facets of CD is its association with various autoimmune and paraneoplastic disorders, including myasthenia gravis (MG) and paraneoplastic pemphigus (PNP) [8-10]. To the best of our knowledge, this case is the first documented instance of SR-HVCD in Saudi Arabia.

## Case presentation

Patient information

We present the case of a 24-year-old Saudi Arabian female previously diagnosed with acetylcholine receptor antibody-positive myasthenia gravis. The patient sought medical attention with complaints of acute-onset shortness of breath and oral thrush. She was subsequently admitted to our institution due to an acute myasthenia gravis exacerbation necessitating intensive care unit (ICU) admission.

Clinical presentation

During the course of her hospitalization, the patient developed conspicuous skin lesions in various anatomical regions, most notably in the oral cavity, bilateral forearms, and hands (both dorsal and palmar aspects). These skin manifestations are presented as flaccid blisters, erosions, crust formation, and erythematous areas. The progressive nature of these lesions warranted further investigation.

Radiological findings

**Figure 1:**
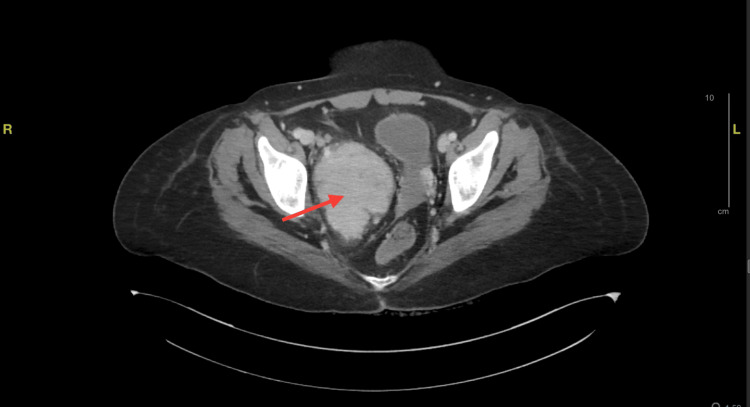
CT scan showing single large abdominal pelvic mass (red arrow), and multiple retroperitoneal and pelvic lymphadenopathy.

A computed tomography (CT) scan of the abdomen was performed, revealing a significant extraperitoneal mass measuring 7.3 cm × 8.7 cm × 6.3 cm in size within the right hemipelvis. This mass exhibited heterogeneous enhancement and contained small foci of calcification. Notably, the scan also detected multiple retroperitoneal and pelvic lymphadenopathies (Figure 1), further adding to the complexity of the case.

Treatment and surgical intervention

Given the patient's clinical presentation and the radiological findings, a multidisciplinary approach was employed. The patient received three cycles of intravenous immunoglobulin (IVIG) therapy to address the acute myasthenia gravis exacerbation. Subsequently, the decision was made to proceed with laparotomy to explore and surgically excise the identified mass.

Histopathological findings

**Figure 2:**
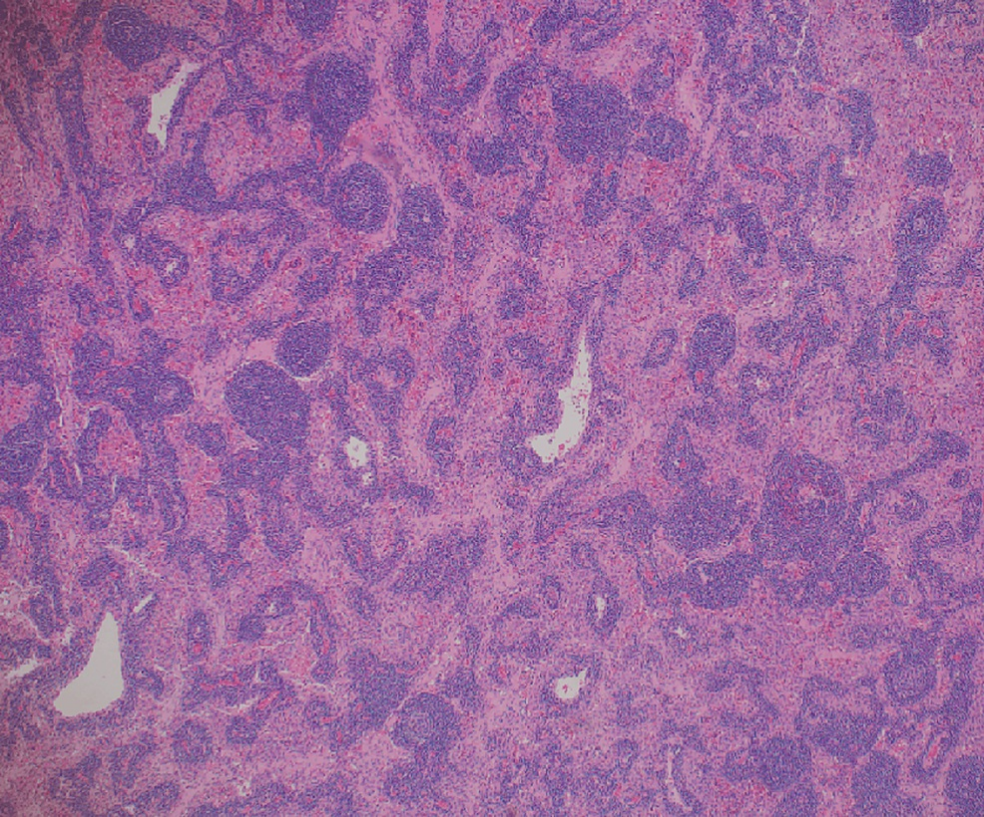
Small lymphoid follicles with expansion of the interfollicular areas by spindle cell proliferation.

**Figure 3:**
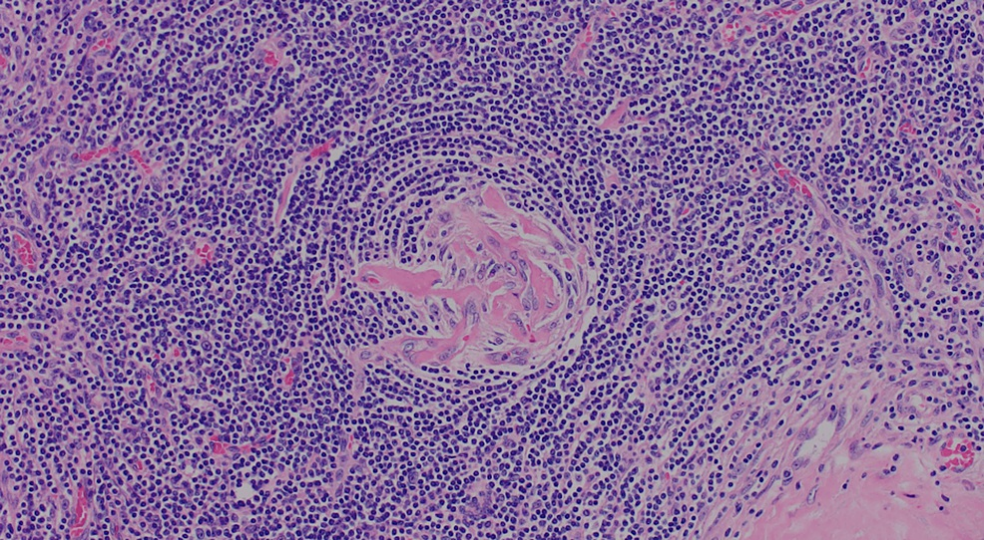
Lymphocytes arranged in concentric rings around the germinal centers, manifesting as an "onion skin” appearance with sclerotic blood vessels "lollipop" appearance.

The histopathological examination of the excised mass revealed a lymphoproliferative lesion characterized by many small follicles with regressed germinal centers (Figure 2). The mantle zone displayed marked expansion with lymphocytes arranged in concentric rings around the germinal centers, manifesting as an "onion skin” appearance. Additionally, some sclerotic blood vessels were noted, radially traversing into some of the germinal centers, imparting a distinctive "lollipop" appearance (Figure 3).

**Figure 4:**
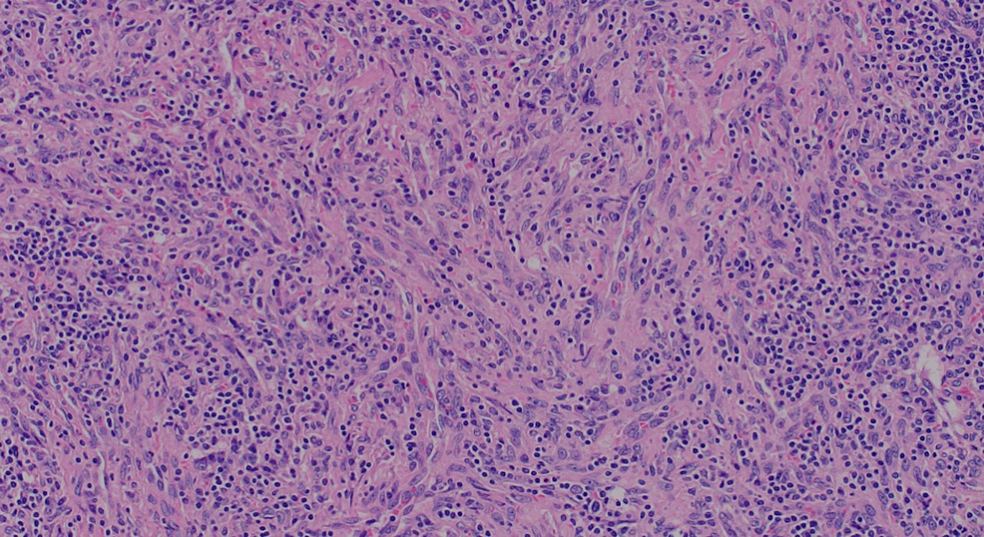
Proliferation of spindle cells, accompanied by an increased number of blood vessels.

**Figure 5:**
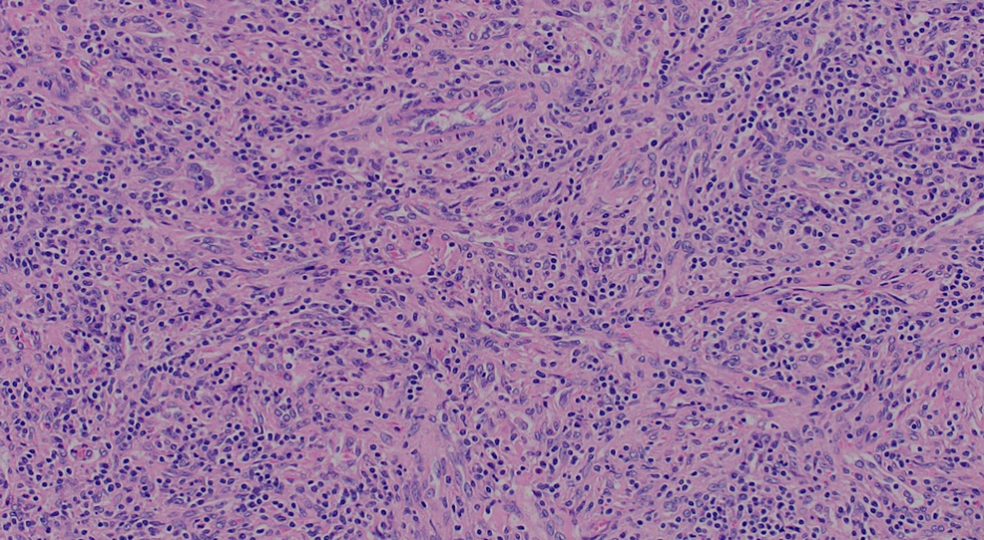
Spindle cell proliferation with extensive lymphocytic infiltrate.

Moreover, the interfollicular areas exhibited substantial proliferation of spindle cells, accompanied by an increased number of blood vessels (Figures 4-5]. The spindle cells displayed mild cytological atypia, focal small visible nucleoli, and slightly irregular nuclear membranes. Mitotic activity was minimal, with no atypical forms identified. No necrotic areas were observed.

Immunohistochemical studies

**Figure 6:**
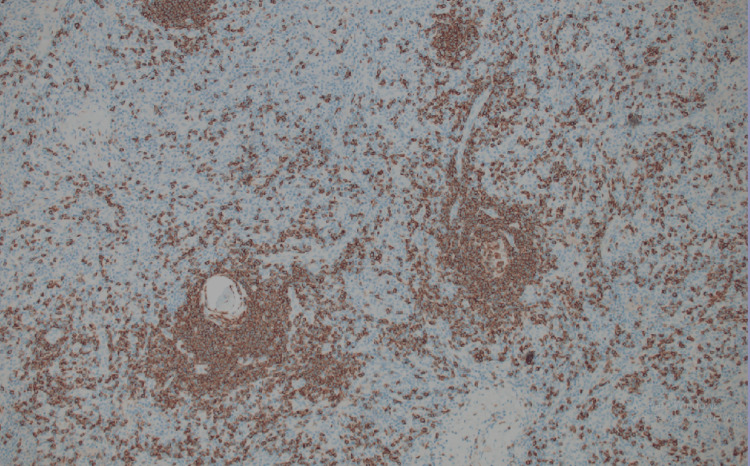
CD20 highlighting strongly positive B-cell in follicular area and interfollicular areas.

**Figure 7:**
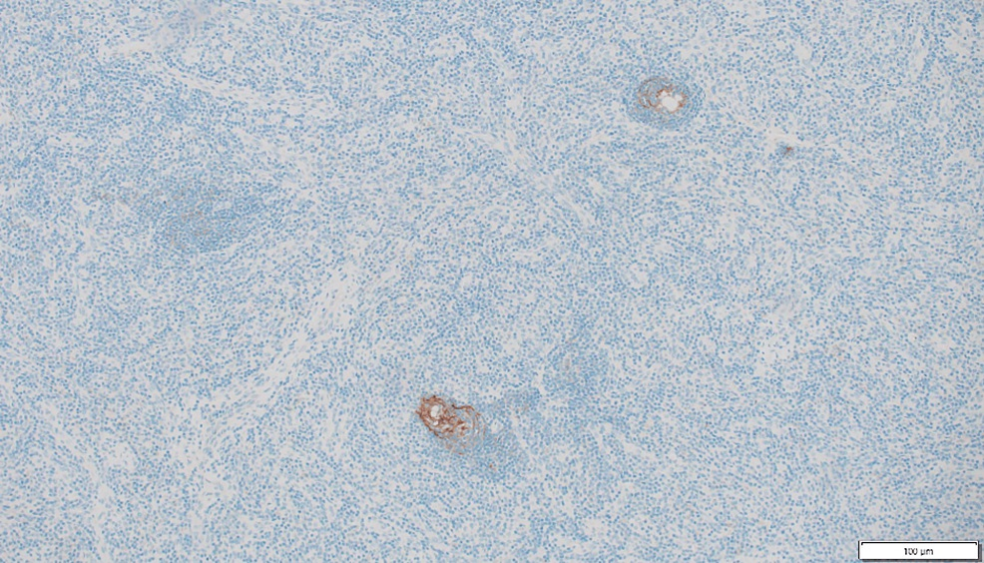
CD21 is negative in interfollicular spindle cell.

**Figure 8:**
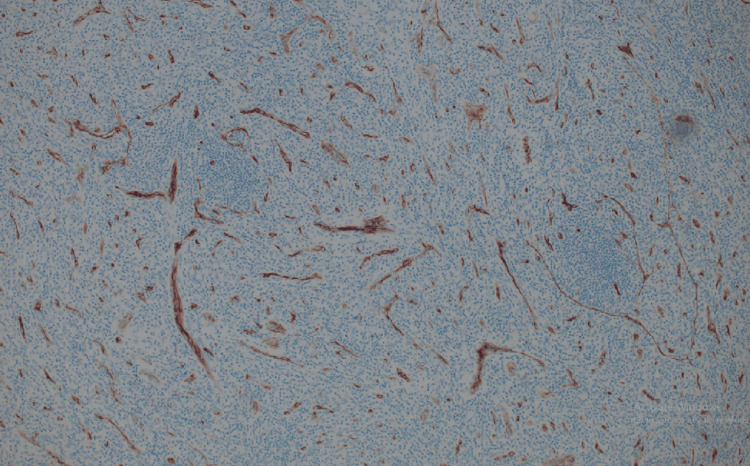
CD34 highlight only vasculature while negative for lymphoid cells.

Immunohistochemical (IHC) studies performed on the specimen yielded valuable insights. The follicles were composed mostly of CD20-positive B lymphocytes (Figure 6). The germinal centers were negative for BCL-2, which is consistent with reactive follicular hyperplasia. The spindle cells in the interfollicular areas were focally positive for smooth muscle actin (SMA) and negative for CD21, S100, Desmin, p63, CK AE1/3, CD31, and CD34 IHC (Figures 7-8). Notably, HHV-8 and TdT IHC were negative.

Diagnosis

The histopathological analysis of the excised mass in our case proved instrumental in confirming the diagnosis of SR-HVCD. The distinctive histological features, including small follicles with regressed germinal centers, concentric rings of lymphocytes, and hyalinized blood vessels, are characteristic of the hyaline vascular subtype of Castleman disease [11,12]. The expansion of interfollicular areas by spindle cells associated with increased vascularity and hyalinized blood vessel walls aligns with the stroma-rich variant of Castleman disease.

The patient's complex clinical presentation, including the association with myasthenia gravis and paraneoplastic pemphigus, underscores the intricate interplay of autoimmune and neoplastic processes.

This case, to the best of our knowledge, marks the first reported instance of a stroma-rich variant of hyaline vascular Castleman disease in Saudi Arabia. It highlights the importance of considering this rare entity in the differential diagnosis of patients presenting with complex clinical pictures and underscores the necessity of multidisciplinary collaboration in the management of such cases.

Patient outcome

After the laparotomic approach, total surgical resection of the mass patient’s skin findings, “paraneoplastic pemphigus,” improved. Hand erosions are completely resolved. Bilaterally, there are multiple violacious brown sclerotic plaques on the palms, along with a few scattered brown macules on the shoulders and thighs. No ulcers, no erosions, and no active lesions were discovered afterward.

## Discussion

CD is a complex and rare group of lymphoproliferative disorders characterized by the abnormal enlargement of lymph nodes or extranodal sites [1,2]. CD exhibits remarkable heterogeneity in clinical presentation, histopathology, and associated disorders. In this context, we offer an in-depth exploration of the different types of CD and their intriguing connections with various autoimmune and paraneoplastic disorders [3-5].

CD can be broadly categorized into two primary classes, UCD and MCD, predicated on the extent of lymph node involvement and clinical manifestations [1,2].

UCD, the more prevalent of the two forms, typically manifests as a localized or solitary mass in a single lymph node region, such as the mediastinum or neck [13,14]. Patients with UCD are mostly asymptomatic or exhibit compression-related symptoms [13-15]. Histologically, UCD can present with hyaline vascular, plasma cell, or mixed subtypes, with the hyaline vascular subtype being the most frequent. The hyaline vascular subtype is characterized by the proliferation of lymphoid follicles with expanded mantle zones that contain one or more regressed germinal centers. Usually, germinal centers are penetrated by hyalinized or sclerotic blood vessels, forming "lollipop-like" hyaline vascular lesions [15-17]. Few of the HVCD cases show marked expansion of the interfollicular area by the florid proliferation of fibroblast-like cells. Those cases were called SR-HVCD [7]. The plasmacytic/mixed subtype is characterized by a well-preserved lymph node with follicular hyperplasia and numerous mature plasma cells expanding the interfollicular area.

In contrast, MCD involves multiple lymph nodes and/or extranodal sites, resulting in a spectrum of systemic symptoms and laboratory abnormalities [5]. These patients frequently present with constitutional symptoms like fever, night sweats, weight loss, fatigue, generalized lymphadenopathy, and hepatosplenomegaly [18-20]. MCD can induce a severe inflammatory state, occasionally culminating in life-threatening complications. Histologically, MCD is often associated with the plasma cell variant, marked by prominent interfollicular plasma cell infiltrates. The mixed subtype, combining features of hyaline vascular and plasma cell variants, can also manifest in MCD [5,18,19].

CD is associated with various autoimmune and paraneoplastic disorders, including MG and PNP [8-10].

In particular, the multicentric form of Castleman disease has been linked to the development or exacerbation of MG [21]. This association is thought to be mediated by the production of proinflammatory cytokines, such as interleukin-6 (IL-6), within CD-affected lymphoid tissue [10]. IL-6 may upregulate acetylcholine receptors at the neuromuscular junction, intensifying MG symptoms. The coexistence of Castleman disease and MG can result in intricate clinical presentations, necessitating comprehensive evaluation and tailored management strategies [21,22].

PNP, a rare and severe autoimmune blistering disorder characterized by painful mucocutaneous lesions, often resembling pemphigus vulgaris but more widespread, is frequently associated with Castleman disease and other malignancies [23]. PNP is believed to result from autoantibodies generated against antigens expressed by both the neoplasm and epithelial tissues [24]. The cross-reactivity of these autoantibodies with epithelial antigens leads to the formation of blistering skin lesions [24,25]. In a study done on the Chinese population, most patients with SR-HVCD had PNP. MG shows no significant difference in prevalence between SR-HVCD and conventional HVCD [26]. In our case, the development of skin lesions in the oral cavity and on the extremities was a critical diagnostic clue. These lesions, resembling flaccid blisters, erosions, crusts, and erythema, were closely aligned with the clinical presentation of PNP. The co-occurrence of Castleman disease and PNP emphasizes the importance of thorough evaluation in patients presenting with complex dermatological manifestations.

Understanding the intricate relationship between Castleman disease and these associated autoimmune and paraneoplastic disorders is pivotal for comprehensive patient care. This case report will illuminate the unique presentation of CD and its associations, offering insights within the context of Saudi Arabia.

## Conclusions

In summary, we have presented the first documented case of SR-HVCD in Saudi Arabia. The unique clinical presentation, marked by the association with MG and PNP, underscores the complexity of CD and its potential to manifest as a paraneoplastic syndrome. This case highlights the critical role of multidisciplinary collaboration in the diagnosis and management of complex cases. It emphasizes the need for heightened clinical suspicion when encountering patients with autoimmune disorders and unusual dermatological manifestations, particularly when MG is coexistent.

Moreover, our case underscores the importance of early intervention in CD to prevent potential complications and improve patient outcomes. Further research into the mechanisms underlying the association between CD and autoimmune disorders is warranted to enhance our understanding of the pathophysiological processes at play.
